# The association between depressive symptoms and self-rated health among university students: a cross-sectional study in France and Japan

**DOI:** 10.1186/s12888-020-02948-8

**Published:** 2020-11-23

**Authors:** Mami Ishida, Ilaria Montagni, Keiichi Matsuzaki, Tomonari Shimamoto, Tanguy Cariou, Takashi Kawamura, Christophe Tzourio, Taku Iwami

**Affiliations:** 1 Department of Preventive Services, Kyoto University School of Public Health, Yoshida-honmachi, Sakyo-ku, Kyoto, 606-8501 Japan; 2 Team HEALTHY, UMR 1219, University of Bordeaux, Inserm, Bordeaux Population Health Research Center, F-33000 Bordeaux, France; 3 Kyoto University Health Service, Yoshida-honmachi, Sakyo-ku, Kyoto, 606-8501 Japan

**Keywords:** Depression, Self-rated health, Students, Sex, Cross-country comparison, Health conditions

## Abstract

**Background:**

Depressive disorders in University students have risen dramatically in the past few decades to the extent that students’ mental health has become a current global public health priority. Obtaining information from University students about their mental health is challenging because of potential embarrassment of disclosing one’s concerns and fear of stigmatization. Self-rated health might be a good solution to evaluate mental health state by a simple and neutral indicator. The aim of the study is to investigate the association between depressive symptoms and self-rated health by sex among University students in France and Japan.

**Methods:**

A cross-sectional study was conducted by using two large cohorts of students aged ≥18 years (*n* = 5655 in Bordeaux, France and *n* = 17,148 in Kyoto, Japan). Depressive symptoms (PHQ-2 scale), Likert scale of self-rated health, socio-demographic characteristics and health habits were collected through self-administered questionnaires. Multivariate logistic regression models were performed to describe the association between depressive symptoms and other variables including self-rated health, stratified by sex.

**Results:**

A high score of PHQ-2 (high depressive symptoms) was associated with poor self-rated health in both cohorts independently of all other variables (OR 2.82, 95%CI 1.99–4.01 in France, OR 7.10, 95%CI 5.76–8.74 in Japan). Although the prevalence of depressive symptoms between sexes was different in French students (males 15.4%, females 25.0%, *p* < 0.001), it was similar in Japanese students (males 3.5%, females 3.3%, *p* = 0.466), who reported less depressive symptoms than French students. The association between depressive symptoms and poor self-rated health was greater in Japanese females (OR 12.40, 95%CI 7.74–20.00) than in males (OR 6.30, 95%CI 4.99–7.95), whereas the strength of the association was almost similar in French students (OR 2.17, 95%CI 0.86–5.47 in males, OR 2.98, 95%CI 2.03–4.38 in females).

**Conclusions:**

Depressive symptoms were associated with self-rated health among University students in both countries with slightly differences in sex. Self-rated health would be a simple, reliable and universal indicator for healthcare professionals and University staff to identify students at risk of depression.

**Supplementary Information:**

The online version contains supplementary material available at 10.1186/s12888-020-02948-8.

## Background

Depressive disorders among University students have risen dramatically in the past few decades to the extent that students’ mental health has become a current global public health priority [1, 2]. University is a time of transition from adolescence to adulthood with drastic lifestyle changes. Students face a new, competitive and challenging environment, and experience stress due to various factors, including pressure to perform academically [3], financial problems, and the burden of autonomy [4]. Suicide is the first cause of death among young adults in Japan [5] and the second in France [6]. A lot of attention has been devoted by policy-makers, mental health professionals, University staff and the media to mental health on University campuses across Europe [7], Northern America [8, 9], Oceania [10] and Asia [11].

Recently, the WHO World Mental Health Survey (WMH) Initiative [12] has collected data of students aged 18–22 years in 21 high-income countries (e.g. France, Northern Ireland) excluding Japan, upper-middle-income countries (e.g. Mexico, Romania) and low- and lower-middle-income countries (e.g. Colombia, Nigeria, People’s Republic of China) [13]. According to this survey, major depressive disorders affect 4.5–7.7% of undergraduates. Differences (~ 10%) in the prevalence of depressive symptoms were observed across participating countries, but overall findings indicated that they were widely diffused among students worldwide. It has become increasingly important for Universities to collect these data in order to investigate what mental health problems affect students, consequently determine which preventive interventions should be implemented, and finally offer appropriate care. Despite this global survey, data on students’ health and wellbeing are still sparsely available and not systematically collected, and comparative studies investigating specific associations of mental health problems are rare especially between Europe and Asia [14]. Both France and Japan are high-income countries, but they have very different cultures and environmental conditions concerning University students, such as university curriculum, legal drinking age, proportion of smoking students, and living environment (living with partner/family members/alone). Similar frequency of distress among students of these two countries might reinforce the idea that students’ mental health deserves global attention, independently from the nation and the culture.

Obtaining information from University students about their mental health is challenging because of potential embarrassment in disclosing one’s concerns and fear of stigmatization [15]. Therefore, it is important to evaluate if simple and more neutral questions could be used as a proxy for mental health state. Self-rated health is a good candidate since it is usually measured with a unique subjective question on a Likert scale, i.e. “How would you rate your health in general?” with the answer options “very good,” “good,” “fair”, “bad” and “very bad” [16]. This measure has been widely used in public health studies [17, 18], including among young adults and students [4, 19]. Through a single subjective question, self-rated health provides a convenient and quick method of assessing reliably an individual’s health status where health, as officially defined by the World Health Organization, is a state of complete physical, mental, and social well-being, not merely the absence of disease or infirmity. Low self-rated health has been closely linked with mortality, even when adjusted for other predictive factors (e.g. health-related behavior, socio-demographic characteristics and objective health status) [16]. Specifically for young adults, self-rated health might be a valid indicator of an individual’s mental health status, independently associated with physical health status (e.g., body weight) and health behaviors including smoking, drinking and sleep [4, 19].

Mental health problems are twice as common in women as in men across most socio-economic contexts and regions [20, 21]. However, some recent cohort studies have shown that differences in reporting depressive symptoms between sexes were not very large, probably because of the new changed role of the woman in the society who is less submitted than in the past [21]. Specifically for University students, results are controversial [22–24], but it was suggested that female students are more inclined towards a more negative view of their health than male students [22]. Therefore, sex is a potentially major confounder in analyses related to mental health.

The purposes of the current comparative study were (1) to investigate the association between depressive symptoms and self-rated health by sex in two large cohorts of University students from two different countries, France and Japan, and (2) to assess whether self-rated health could be used as a simple indicator for screening University students with potential depressive symptoms across countries. We hypothesized that lower levels of self-rated health would be significantly associated with higher depressive symptoms. As a result, making a simple question on self-rated health would allow detecting students in distress, without using larger and more intrusive psychological assessment scales.

## Methods

This is a cross sectional study using data from two independent cohorts of University students: i-Share in France and the Kyoto University students’ cohort in Japan.

### The i-share cohort study

The i-Share project (Internet-based Students Health Research Enterprise, www.i-share.fr) is an ongoing prospective population-based cohort study on the health and wellbeing of French University students. The Universities of Bordeaux and Versailles Saint-Quentin were the first higher education institutes to recruit voluntary participants from February 2013; other French Universities have further joined the project. Inclusion criteria are: being ≥18 years and able to read and understand French. Recruitment strategies comprise the use of flyers, information stands at University campuses, classroom-based interventions, e-mailing and social media. The i-Share project is based on an online questionnaire containing items on physical and mental health (including self-rated health), socio-demographic characteristics and healthy habits. Three months after the completion of this questionnaire, a supplementary online questionnaire focused on mental health status is addressed to students. This questionnaire, which is still completed on a voluntary basis, asks specific information on depressive symptoms. For this comparative study, we used data available as of September 3rd, 2018. The i-Share project is carried out in accordance with the Declaration of Helsinki and has been approved by the National Commission of Informatics and Liberty (Commission Nationale de l’Informatique et des Libertés - CNIL), number DR-2013-019. Every student is requested to sign an online informed consent form before completion of the baseline questionnaire, according to the Committee of Protection of Persons (Comités de Protection des Personnes, CPP). No further local ethical approval was needed [25].

### The Kyoto University students’ cohort

Kyoto University Health Service carries out an annual health checkup addressed to all students in April. This checkup consists of anthropometric measures including height, weight, blood pressure, urinalysis, and a self-administrated web-based questionnaire including items about lifestyle, physical and mental health status, past and present medical history, sleep satisfaction and self-rated health. Concerned students come from 10 undergraduate school departments and 20 graduate school departments covering all fields of study such as humanities, sciences, health studies, law and economy. Data for this comparative study refer to the year 2017. We excluded all students who answered the web-based questionnaire, but did not participate in the annual health checkup. All analyses and procedures were anonymous and in accordance with the Declaration of Helsinki and approved by the Institutional Review Board of the Kyoto University Ethical Committee (Ethics Code No: R1732). Individual informed consent was waived for the use of de-identified clinical data according to the national ethical guidelines of Japan.

### Outcome, exposure and confounding variables

#### Depressive symptoms

Depressive symptoms were assessed using the first two items of the 9-item Patient Health Questionnaire (PHQ-9) [26], also known as the PHQ-2 [27]. These two items enquiry about the frequency of the symptoms of depressed mood (feeling down, depressed or hopeless) and anhedonia (little interest or pleasure in doing things), scoring each as 0 (not at all), 1 (several days), 2 (more than half the days) and 3 (nearly every day). Thus, the total score of the PHQ-2 can range from 0 to 6. As previously recommended [28], we used the cutoff score of 3, by dichotomizing this variable into two categories: “low depressive symptoms” (0–2) and “high depressive symptoms” (3–6). PHQ-2 has demonstrated good reliability and validity to assess major depression among primary care patients [27].

#### Self-rated health

To assess health status, we used a five-point Likert scale of self-rated health corresponding to the question “How would you rate your health in general?”. Answers were “very good,” “good,” “fair”, “bad” or “very bad” [16]. This variable was further dichotomized into two categories: “poor” (including “bad” or “very bad”) and “good” (including “fair”, “good” or “very good”) self-rated health.

#### Socio-demographic characteristics

We collected the following variables: sex (male, female), age (quantitative variable), and year of study (the first year of undergraduate versus other years).

#### Sleep quality

In the i-Share cohort, sleep quality was assessed through a single item from the Pittsburgh Sleep Quality Index [29]. Students were asked to categorize their sleep quality during the three previous months preceding the survey as “good”, “somewhat good”, “neither good nor bad”, “somewhat bad” or “bad”. In the Kyoto cohort, sleep quality was assessed by categorizing sleep satisfaction as “very satisfied”, “satisfied”, “dissatisfied”, or “very dissatisfied”. In order to compare the results from these two different questions, we further dichotomized the answer options from the two questionnaires into two categories: “bad” (including “somewhat bad” or “bad” for i-Share, and “dissatisfied” or “very dissatisfied” for Kyoto) and “good” (including “good”, “somewhat good” or “neither good nor bad” for i-Share, and “very satisfied” or “satisfied” for Kyoto) sleep.

#### Body mass index (BMI)

In the i-Share cohort, height and weight were self-reported in the web-based baseline questionnaire. In the Kyoto cohort, when having health checkup, students’ height and weight were measured. In both cohorts, we calculated body mass index (BMI) as weight in kg divided by height in m squared (kg/m^2^) and categorized it in four groups: underweight (< 18.5), normal weight (≥18.5, < 25), overweight (≥25.0, < 30), and obesity (≥30).

#### Health habits

In the i-Share cohort, students were asked whether they were regularly practicing one or more sport activities (“yes” or “no”). In the Kyoto cohort, students were asked how often they usually exercised in the last month (“everyday”, “sometimes, or “rarely”). The physical activity categories were dichotomized into “regular” (corresponding to “yes” in i-Share, “everyday” or “sometimes” in Kyoto), and “occasional or less” (corresponding to “no” in i-Share and “rarely” in Kyoto). For smoking status, in both studies the categories were dichotomized into “yes” (corresponding to “daily or occasionally smoking”) and “no” (corresponding to “never smoking”). Alcohol consumption was assessed as current frequency of consumption of alcoholic beverages (i.e. beer, wine, whisky, vodka, tequila, cocktails). More precisely, in the i-Share questionnaire the categories were divided into “never”, “from once a year to once per month”, “from several times per month to once per week”, and “several times per week”. In the Kyoto questionnaire, the categories were divided into “never”, “sometimes”, “20 g/day” and “with trouble”. Finally, to compare data from the two studies, the categories for alcohol consumption were dichotomized into “never” and “at least sometimes” drinking.

### Statistical analysis

We performed general descriptive statistics for each cohort and calculated comparative *p* values between the two cohorts. Chi-square test for categorical variables and t test for continuous variables were used respectively. Then, we performed the same description by the two categories of depressive symptoms (0–2 and 3–6 scores) in order to obtain the univariate associations with all covariates, including self-rated health. Multivariate logistic regression models were performed for each cohort to further explore the association between depressive symptoms and all covariates, including self-rated health. For each multivariate logistic regression model, we tested potential cofounding factors (interactions) and/or effect modifiers (relative variation of the ORs).

Main analyses were performed in male and female students separately. Missing values were included in the model as a separate category. All tests were 2-tailed, and *p* values of < 0.05 were considered as statistically significant. SAS version 9.4 (SAS Institute Inc., Cary, NC USA) and R version 3.5.1 for Windows were used for all analyses.

## Results

The French cohort included 5655 students, and the Japanese cohort included 17,148 students.

Table 1 provides descriptive statistics of all variables used in this study per cohort. Descriptive statistics of these variables stratified by sex are presented as a supplementary material in Table S1.

**Table 1:** Description of the study population

Variables	i-Share (France) *n* = 5655	Kyoto (Japan) *n* = 17,148	*P* value
PHQ-2, n (%)			
Low depressive symptoms [0–2]	4355 (77.0)	16,555 (96.5)	< 0.001
High depressive symptoms [3–6]	1300 (23.0)	593 (3.5)	
Self-rated health, n (%)			
Good	5499 (97.2)	16,378 (95.5)	< 0.001
Poor	156 (2.8)	770 (4.5)	
Sex, n (%)			
Female	4457 (78.8)	4193 (24.5)	< 0.001
Male	1198 (21.1)	12,955 (75.5)	
Age, mean (SD)	20.8 (2.6)	22.4 (4.4)	< 0.001
Year of study, n (%)			
1st undergraduate	2214 (39.2)	2698 (15.7)	< 0.001
Other years	3441 (60.8)	14,450 (84.3)	
Sleep, n (%)			
Good	4478 (79.2)	12,740 (74.3)	< 0.001
Bad	1177 (20.8)	4408 (25.7)	
BMI^a^, n (%)			
< 18.5	729 (12.9)	2290 (13.3)	< 0.001
[18.5-24.9]	4030 (71.3)	13,232 (77.2)	
[25–29.9]	471 (8.3)	1381 (8.1)	
> = 30	120 (2.1)	245 (1.4)	
Physical activity, n (%)			
Regular	4950 (87.5)	11,435 (66.7)	< 0.001
Occasional or less	705 (12.5)	5713 (33.3)	
Smoking, n (%)			
No	4245 (75.1)	16,677 (97.3)	< 0.001
Yes	1410 (24.9)	471 (2.7)	
Alcohol consumption, n (%)			
Never	434 (7.7)	7292 (42.5)	< 0.001
At least sometimes	5221 (92.3)	9856 (57.5)	

Abbreviations; *PHQ-2* The 2-item Patient Health Questionnaire, *BMI* Body mass index

^a^i-Share cohort had BMI missing values: 305 (5.4)

Description of the two cohorts concerning PHQ-2, Self-rated heath, Sex, Age, Year of study, Sleep, BMI, Physical activity, Smoking and Alcohol consumption, respectively

High depressive symptoms (PHQ-2 (3–6)) were reported by 23.0% of French students (15.4% for males and 25.0% for females), and by 3.5% of Japanese students (3.5% for males and 3.3% for females). Poor self-rated health was reported by 2.8% of French students (2.3% for males and 2.9% for females), and by 4.5% of Japanese students (5.0% for males and 2.9% for females). Other major differences between countries concerned regular physical activity (87.5% of French students versus 66.7% of Japanese students), smoking (24.9% of French students versus 2.7% of Japanese students), and alcohol consumption (92.3% of French students versus 57.5% of Japanese students).

Table 2 presents the univariate associations between PHQ-2 score and all other variables including self-rated health. Results stratified by sex are presented as a supplementary material in Table S2.

**Table 2:** Univariate comparison of all variables according to PHQ-2 score in the two cohorts

Variables	i-Share (France)			Kyoto (Japan)		
	PHQ-2			PHQ-2		
	[0–2]	[3–6]	*p* value	[0–2]	[3–6]	*p* value
	*n* = 4355	*n* = 1300		*n* = 16,555	*n* = 593	
Self-rated health, n (%)						
Good	4280 (98.3)	1219 (93.8)	< 0.001	15,973 (96.5)	405 (68.3)	< 0.001
Poor	75 (1.7)	81 (6.2)		582 (3.5)	188 (31.7)	
Sex, n (%)						
Female	3342 (76.6)	1115 (85.8)	< 0.001	4056 (24.5)	137 (23.1)	0.466
Male	1013 (23.3)	185 (14.2)		12,499 (75.5)	456 (76.9)	
Age, mean (SD)	20.9 (2.6)	20.4 (2.3)	< 0.001	22.4 (4.4)	22.7 (3.4)	0.064
Year of study, n (%)						
1st undergraduate	1616 (37.1)	598 (46.0)	< 0.001	2662 (16.1)	36 (6.1)	< 0.001
Other years	2739 (62.9)	702 (54.0)		13,893 (83.9)	557 (93.9)	
Sleep, n (%)						< 0.001
Good	3618 (83.1)	860 (66.2)	< 0.001	12,506 (75.5)	234 (39.5)	
Bad	737 (16.9)	440 (33.8)		4049 (24.5)	359 (60.5)	
BMI^a^, n (%)						
< 18.5	532 (12.2)	197 (15.2)	< 0.001	2202 (13.3)	88 (14.8)	< 0.001
[18.5–24.9]	3159 (72.5)	871 (67.0)		12,793 (77.3)	439 (74.0)	
[25–29.9]	363 (8.3)	108 (8.3)		1336 (8.1)	45 (7.6)	
> =30	80 (1.8)	40 (3.1)		224 (1.4)	21 (3.5)	
Physical activity, n (%)						
Regular	3820 (87.7)	1130 (86.9)	0.448	11,124 (67.2)	311 (52.4)	< 0.001
Occasional or less	535 (12.3)	170 (13.1)		5431 (32.8)	282 (47.6)	
Smoking, n (%)						
No	3296 (75.7)	949 (73.0)	0.049	16,109 (97.3)	568 (95.8)	0.039
Yes	1059 (24.3)	351 (27.0)		446 (2.7)	25 (4.2)	
Alcohol consumption, n (%)						
Never	300 (6.9)	134 (10.3)	< 0.001	7029 (42.5)	263 (44.4)	0.375
At least sometimes	4055 (93.1)	1166 (89.7)		9526 (57.5)	330 (55.6)	

Abbreviation; PHQ-2, the 2-item Patient Health Questionnaire; *BMI* Body mass index

^a^i-Share cohort had BMI missing values: 221 (5.1) for [0–2] and 84 (6.5) for [3–6]; *p* value was calculated without missing values

There was a similar pattern of association between PHQ-2 and self-rated health in both cohorts. Poor self-rated health was significantly and positively associated with high depressive symptoms in both French and Japanese students (*p* < 0.001). 6.2% of French students with high depressive symptoms (PHQ-2 score (3–6)) and 1.7% of students with low depressive symptoms (PHQ-2 score (0–2)) reported poor self-rated health. On the other hand, 31.7% of Japanese students with high depressive symptoms (PHQ-2 score (3–6)) and 3.5% of students with low depressive symptoms (PHQ-2 score (0–2)) reported poor self-rated health. Concerning the detection of depressive symptoms by self-rated health, sensitivity was 6.2 and 31.7% in French and Japanese students, and specificity was 98.3 and 96.5% in French and Japanese students, respectively. Similarly, bad sleep quality, BMI < 18.5, BMI ≥ 30 and smoking habit were associated with high depressive symptoms in both cohorts with *p* values comprised between 0.049 and < 0.001. Never drinking alcohol was associated with high depressive symptoms only among French students (*p* < 0.001).

On the other hand, the two cohorts reported different patterns of association between PHQ-2 and sex, age, year of study, and physical activity. Female sex was associated with high depressive symptoms in the French cohort (*p* < 0.001), whereas there was no sex difference in the Japanese cohort (*p* = 0.466). French students with high depressive symptoms were younger than those with low depressive symptoms (*p* < 0.001), whereas Japanese students with high depressive symptoms were older although the association was not significant (*p* = 0.064). Moreover, the 1st undergraduate year was associated with high depressive symptoms in French students (*p* < 0.001), whereas the 1st undergraduate year was associated with low depressive symptoms in Japanese students (*p* < 0.001). Less physical activity was associated with high depressive symptoms in Japanese students (*p* < 0.001), whereas this association was not significant in French students (*p* = 0.448).

Finally, Table 3 illustrates the results of the multivariate logistic regression analyses. Results stratified by sex are presented in Table S3.

**Table 3:** Multivariate logistic regression analysis

Variables	i-Share (France) *n* = 5350			Kyoto (Japan) *n* = 17,148		
	Odds Ratio	95% wald CI	*p* value	Odds Ratio	95% wald CI	*p* value
Self-rated health, n (%)						
Good	Ref	–	< 0.001	Ref	–	< 0.001
Poor	2.82	1.99–4.01		7.10	5.76–8.74	
Sex, n (%)						
Female	Ref	–	< 0.001	Ref	–	0.681
Male	0.61	0.51–0.73		0.96	0.78–1.18	
Age, mean (SD)	0.95	0.92–0.98	0.001	1.00	0.97–1.02	0.645
Year of study, n (%)						
1st undergraduate	Ref	–	0.056	Ref	–	< 0.001
Other years	0.85	0.73–1.00		2.40	1.65–3.48	
Sleep, n (%)						
Good	Ref	–	< 0.001	Ref	–	< 0.001
Bad	2.30	1.98–2.67		2.94	2.44–3.53	
BMI, n (%)						
< 18.5	1.20	1.00–1.45	0.075	1.03	0.80–1.32	0.015
[18.5–24.9]	Ref	–		Ref	–	
[25–29.9]	1.07	0.85–1.36		0.90	0.65–1.25	
> =30	1.48	0.99–2.21		2.21	1.34–3.64	
Physical activity, n (%)						
Regular	Ref	–	0.592	Ref	–	0.001
Occasional or less	1.06	0.87–1.29		1.35	1.13–1.61	
Smoking, n (%)						
No	Ref	–	0.049	Ref	–	0.094
Yes	1.16	1.00–1.35		1.46	0.94–2.26	
Alcohol consumption, n (%)						
Never	Ref	–	0.003	Ref	–	< 0.001
At least sometimes	1.42	1.12–1.80		1.44	1.20–1.73	

Abbreviation; *BMI* Body mass index

Association between PHQ-2 and Self-rated health, adjusted for Sex, Age, Year of study, Sleep, BMI, Physical activity, Smoking and Alcohol consumption

Poor self-rated health was significantly associated with high depressive symptoms in both cohorts although the level of the point estimate was higher in Japanese students (i-Share: OR 2.82, 95%CI 1.99–4.01, *p* < 0.001, Kyoto: OR 7.10, 95%CI 5.76–8.74, *p* < 0.001). In addition, bad sleep quality (i-Share: OR 2.30, 95%CI 1.98–2.67, *p* < 0.001, Kyoto: OR 2.94, 95%CI 2.44–3.53, *p* < 0.001), BMI ≥ 30 (i-Share: OR 1.48, 95%CI 0.99–2.21, *p* = 0.075, Kyoto: OR 2.21, 95%CI 1.34–3.64, *p* = 0.015) and never drinking alcohol (i-Share: OR 1.42, 95% CI 1.12–1.80, *p* = 0.003, Kyoto: OR 1.44, 95%CI 1.20–1.73, *p* < 0.001) were also associated with high depressive symptoms in both cohorts. Smoking was not significantly associated with PHQ-2 in both cohorts.

Female sex was associated with high depressive symptoms in French students (OR 0.61, 95%CI 0.51–0.73, *p* < 0.001), but not in Japanese students (OR 0.96, 95%CI 0.78–1.18, *p* = 0.681). French students with high depressive symptoms were younger (OR 0.95, 95%CI 0.92–0.98, *p* = 0.001), whereas there was no association between age and PHQ-2 in Japanese students (OR 1.00, 95%CI 0.97–1.02, *p* = 0.645). In addition, years other than the 1st undergraduate year were associated with high depressive symptoms in Japanese students (OR 2.40, 95%CI 1.65–3.48, *p* < 0.001), whereas 1st undergraduate year was associated with high depressive symptoms in French students (OR 0.85, 95%CI 0.73–1.00, *p* = 0.056) although the association was not significant. BMI < 18.5 was associated with high depressive symptoms in French students (OR 1.20, 95%CI 1.00–1.45, *p* = 0.075), whereas not in Japanese students (OR 1.03, 95%CI 0.80–1.32, *p* = 0.015). Less physical activity was associated with high depressive symptoms in Japanese students (OR 1.35, 95%CI 1.13–1.61, *p* = 0.001), but not in French students (OR 1.06, 95%CI 0.87–1.29, *p* = 0.592).

As shown in Table S3, there were some different results depending on sex. Among French males, no significant association between PHQ-2 and self-reported health was identified (OR 2.17, 95%CI 0.86–5.47, *p* = 0.100). The correlation between self-rated health and depressive symptoms, as well as the score of PHQ-2 and the numbers and percentages of students with elevated depressive symptoms in each five categories of self-rated health are reported as Supplemental Material.

Finally, since age and year of study were correlated, we performed a sensitive analysis in both cohorts by running models with either age or year of study, and we observed that results were the same across all models. Moreover, since we found different interactions in each cohort, we performed another sensitive analysis to take interactions into models respectively and confirmed that the final results did not change.

## Discussion

In the two large cohorts of French and Japanese University students, we found that high depressive symptoms and poor self-rated health were strongly associated, independently from all measured sociodemographic conditions and health habits. There were major differences between cohorts including prevalence of depressive symptoms (23.0% in French students and 3.5% in Japanese students) and other cultural and socio-demographic characteristics as well as health habits. Despite these differences, the association between depressive symptoms and poor self-rated health was persistent after adjustment on several potential confounders. We interpreted self-rated health as an important common component factor of depressive symptoms in University students by these robust results of the association in two large cohorts with completely different characteristics.

We could interpret the marked difference of depressive symptoms in the two cohorts as follows. Cultural and social differences between the two countries may be part of the explanation as well as specific characteristics of the participants of each sample. Disclosure of mental health problems could be easier in Western countries compared to Japan [30] where stigma toward mental health is still relatively strong [31]. French students were included on a voluntary basis while Japanese students were included in a compulsory manner, and unmeasured potential participation bias could lead to an over-representation of mental health issues in French students. However, other studies among students in Western countries found a higher prevalence of mental health problems than among Japanese students [3, 24].

We took into particular consideration the role of sex in reporting depression and self-rated health. Our results showed that sex difference in the depression prevalence was inconsistent in the two cohorts (15.4% for males and 25.0% for females in French students, 3.5% for males and 3.3% for females in Japanese students). Likewise, as far as self-rated health is concerned, there was no sex difference in French students (2.3% for males and 2.9% for females), whereas slightly more Japanese male students reported poor self-rated health compared to female students (5.0% for males and 2.9% for females). This finding was in line with previous studies showing that self-rated health had yielded mixed findings on the role of sex [4, 22–24]. In view of these controversial results and the opposite distribution of females in our study (78.8% in the French cohort versus 24.5% in the Japanese cohort), we considered it important to explore how sex could influence the association between depressive symptoms and self-reported health. Female sex more strongly affected the association between poor self-rated health and high depressive symptoms in Japanese students as shown in a previous study [4]. This indicated that self-rated health could be a more sensitive marker of mental disease in females, although this finding was not identified in French students. Regarding under-enrollment of female students in Japan, it might represent a socio-political issue linked to gender discrimination [32], as gender ratio of University enrollment is still great. We can hypothesize that Japanese female students reported less poor self-rated health and less high depressive symptoms compared to French students partially because females of the Japanese sample might be more resilient and more supported by their family and surroundings than average females.

Besides self-rated health, sleep and alcohol consumption were the sole factors remaining significantly associated with the PHQ-2 in France and Japan. Because self-rated health is a simple subjective question, self-rated health could partially reflect health behavior. Sleep and alcohol consumption, for instance, might consist self-rated health as components of health behavior, but they are also considered to be independent high-risk factors of depressive symptoms. These two factors also need particular attention when designing intervention to promote students’ mental health as proved by previous studies [33–36]. Moreover, lifestyle habits such as body weight control [37], exercise [38, 39] and stopping smoking [40, 41] might improve mental health among some students.

Our study has some limitations. First, some questions (sleep quality, physical activity and alcohol consumption) were slightly different between the two cohorts and recategorizing variables might slightly impact on final results. However, sleep quality and alcohol consumptions reported similar effects on depressive symptoms. Second, France and Japan are two very different countries in terms of culture, politics, living conditions, etc. Therefore, there were probably still unmeasured confounders we could not adjust on in our analysis. Third, results might not be generalized to other young adult populations because of representativeness of the participants. The i-Share cohort consists of students who are included on a voluntary basis, whereas the Kyoto cohort consists of all students of a single University which consists of highly educated young people in Japan. However, these issues of representativeness and participation are unlikely to have a strong impact on our main finding. Indeed, the association between depression and self-rated health was not influenced by the measured characteristics of the samples. Regardless of these limitations, our study has the strength of being based on two large ongoing cohorts of students coming from two different countries, in Europe and in Asia, collecting similar information.

## Conclusions

Depressive symptoms were significantly associated with self-rated health among University students of both cohorts despite substantial social, cultural and educational differences between France and Japan. This association was confirmed in students of both male and female students, but it was stronger in female students. Self-rated health would be a simple and universal indicator to be used across countries as a component factor for mental health in University students, especially for female students.

## Supplementary Information

**Additional file 1:** **Table S1.** Description of the study population stratified by Sex. Abbreviations; PHQ-2, the 2-item Patient Health Questionnaire; BMI, body mass index. *i-Share cohort had BMI missing values: male 49(4.0), female 257 (5.8). Description of the two cohorts, stratified by Sex, concerning PHQ-2, Self-rated heatrh, Sex, Age, Year of study, Sleep, BMI, Physical activity, Smoking and Alcohol consumption, respectively. **Table S2.** Univariate comparison of all variables according to PHQ-2 score in the two cohorts stratified by Sex. Abbreviations; PHQ-2, the 2-item Patient Health Questionnaire; BMI, body mass index. *i-Share cohort had BMI missing values: 221 (5.1) for [0–2] and 84 (6.5) for [3–6]; *p* value was calculated without missing values. **Table S3.** Multivariate logistic regression analysis stratified by Sex. Abbreviation; BMI, body mass index. Association between PHQ-2 and Self-rated health, stratified by Sex, adjusted for Sex, Age, Year of study, Sleep, BMI, Physical activity, Smoking and Alcohol consumption. **Figure S1-1.** Correlation between self-rated health and depressive symptoms, PHQ-2 score and numbers and percentages of students with elevated depressive symptoms in each of the five categories of self-rated health: i-Share cohort, Spearman correlation coefficient 0.261, *P* value < 0.001. **Figure S1-2.** Correlation between self-rated health and depressive symptoms, PHQ-2 score and numbers and percentages of students with elevated depressive symptoms in each of the five categories of self-rated health: Kyoto cohort, Spearman correlation coefficient 0.339, *P* value < 0.001.

## Data Availability

The datasets used and/or analyzed during the current study are available from the corresponding author on reasonable request.

## Abbreviations

PHQ-2: The 2-item Patient Health Questionnaire
BMI: Body mass index
