# Supplementary material for: The association between depressive symptoms and self-rated health among university students: a cross-sectional study in France and Japan
Source: BMC Psychiatry. 2020 Nov 23;20:549. doi: 10.1186/s12888-020-02948-8 (PMC7685657; doi:10.1186/s12888-020-02948-8)
Supplement: Supplementary file 1 — Additional file 1: Table S1. Description of the study population stratified by Sex. Abbreviations; PHQ-2, the 2-item Patient Health Questionnaire; BMI, body mass index. *i-Share cohort had BMI missing values: male 49(4.0), female 257 (5.8). Description of the two cohorts, stratified by Sex, concerning PHQ-2, Self-rated heatrh, Sex, Age, Year of study, Sleep, BMI, Physical activity, Smoking and Alcohol consumption, respectively. Table S2. Univariate comparison of all variables according to PHQ-2 score in the two cohorts stratified by Sex. Abbreviations; PHQ-2, the 2-item Patient Health Questionnaire; BMI, body mass index. *i-Share cohort had BMI missing values: 221 (5.1) for [0–2] and 84 (6.5) for [3–6]; p value was calculated without missing values. Table S3. Multivariate logistic regression analysis stratified by Sex. Abbreviation; BMI, body mass index. Association between PHQ-2 and Self-rated health, stratified by Sex, adjusted for Sex, Age, Year of study, Sleep, BMI, Physical activity, Smoking and Alcohol consumption. Figure S1-1. Correlation between self-rated health and depressive symptoms, PHQ-2 score and numbers and percentages of students with elevated depressive symptoms in each of the five categories of self-rated health: i-Share cohort, Spearman correlation coefficient 0.261, P value < 0.001. Figure S1-2. Correlation between self-rated health and depressive symptoms, PHQ-2 score and numbers and percentages of students with elevated depressive symptoms in each of the five categories of self-rated health: Kyoto cohort, Spearman correlation coefficient 0.339, P value < 0.001. [file 12888_2020_2948_MOESM1_ESM.zip › Additional File (Figure)R3.pptx]

## Slide 1
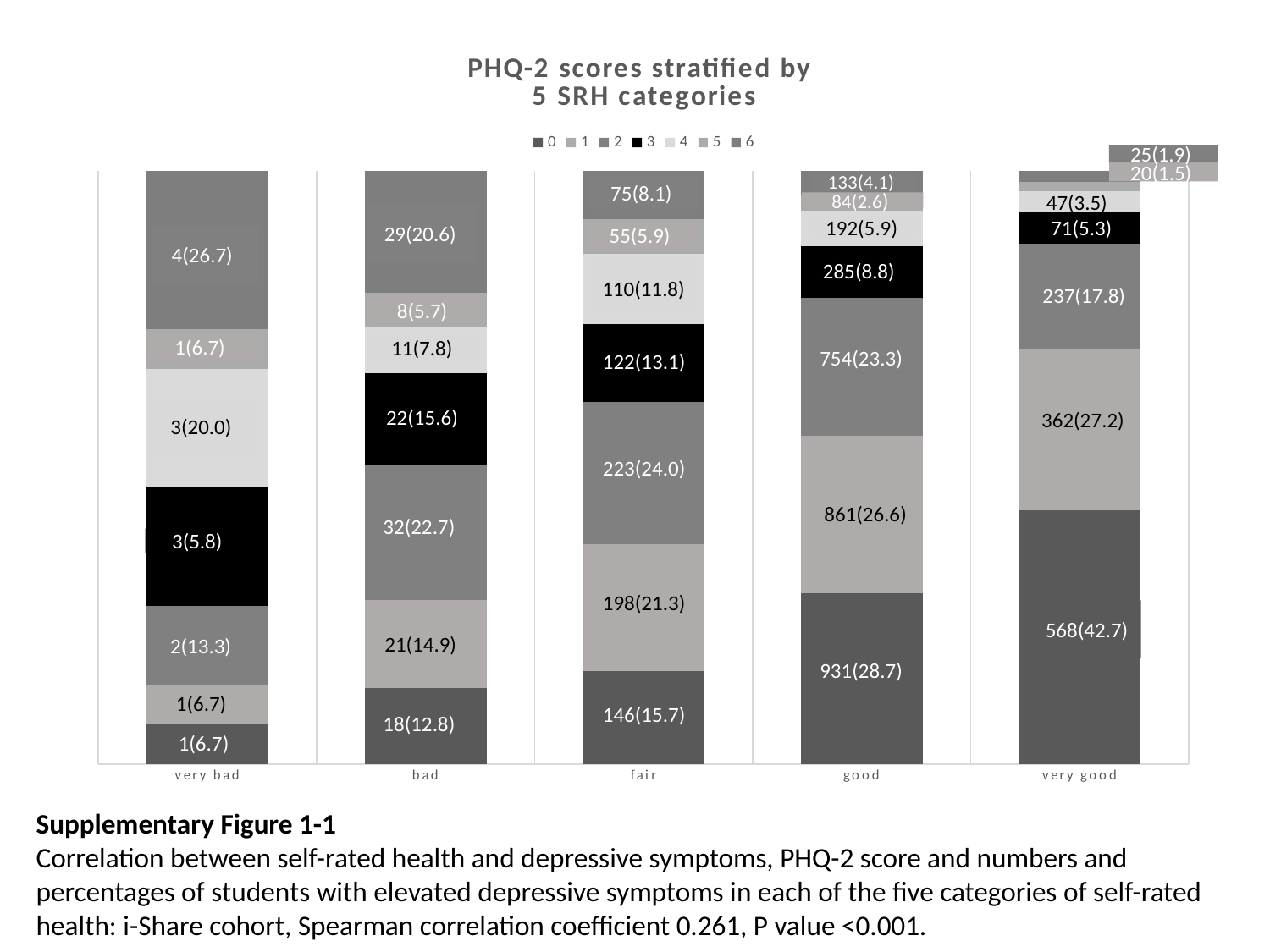

### Chart: PHQ-2 scores stratified by
5 SRH categories
| Category | 0 | 1 | 2 | 3 | 4 | 5 | 6 |
|---|---|---|---|---|---|---|---|
| very bad | 1.0 | 1.0 | 2.0 | 3.0 | 3.0 | 1.0 | 4.0 |
| bad | 18.0 | 21.0 | 32.0 | 22.0 | 11.0 | 8.0 | 29.0 |
| fair | 146.0 | 198.0 | 223.0 | 122.0 | 110.0 | 55.0 | 75.0 |
| good | 931.0 | 861.0 | 754.0 | 285.0 | 192.0 | 84.0 | 133.0 |
| very good | 568.0 | 362.0 | 237.0 | 71.0 | 47.0 | 20.0 | 25.0 |25(1.9)
20(1.5)
133(4.1)
75(8.1)
47(3.5)
84(2.6)
29(20.6)
192(5.9)
71(5.3)
55(5.9)
4(26.7)
285(8.8)
110(11.8)
237(17.8)
8(5.7)
1(6.7)
11(7.8)
754(23.3)
122(13.1)
362(27.2)
3(20.0)
22(15.6)
223(24.0)
861(26.6)
32(22.7)
3(5.8)
198(21.3)
568(42.7)
21(14.9)
2(13.3)
931(28.7)
146(15.7)
1(6.7)
18(12.8)
1(6.7)
Supplementary Figure 1-1
Correlation between self-rated health and depressive symptoms, PHQ-2 score and numbers and percentages of students with elevated depressive symptoms in each of the five categories of self-rated health: i-Share cohort, Spearman correlation coefficient 0.261, P value <0.001.

## Slide 2
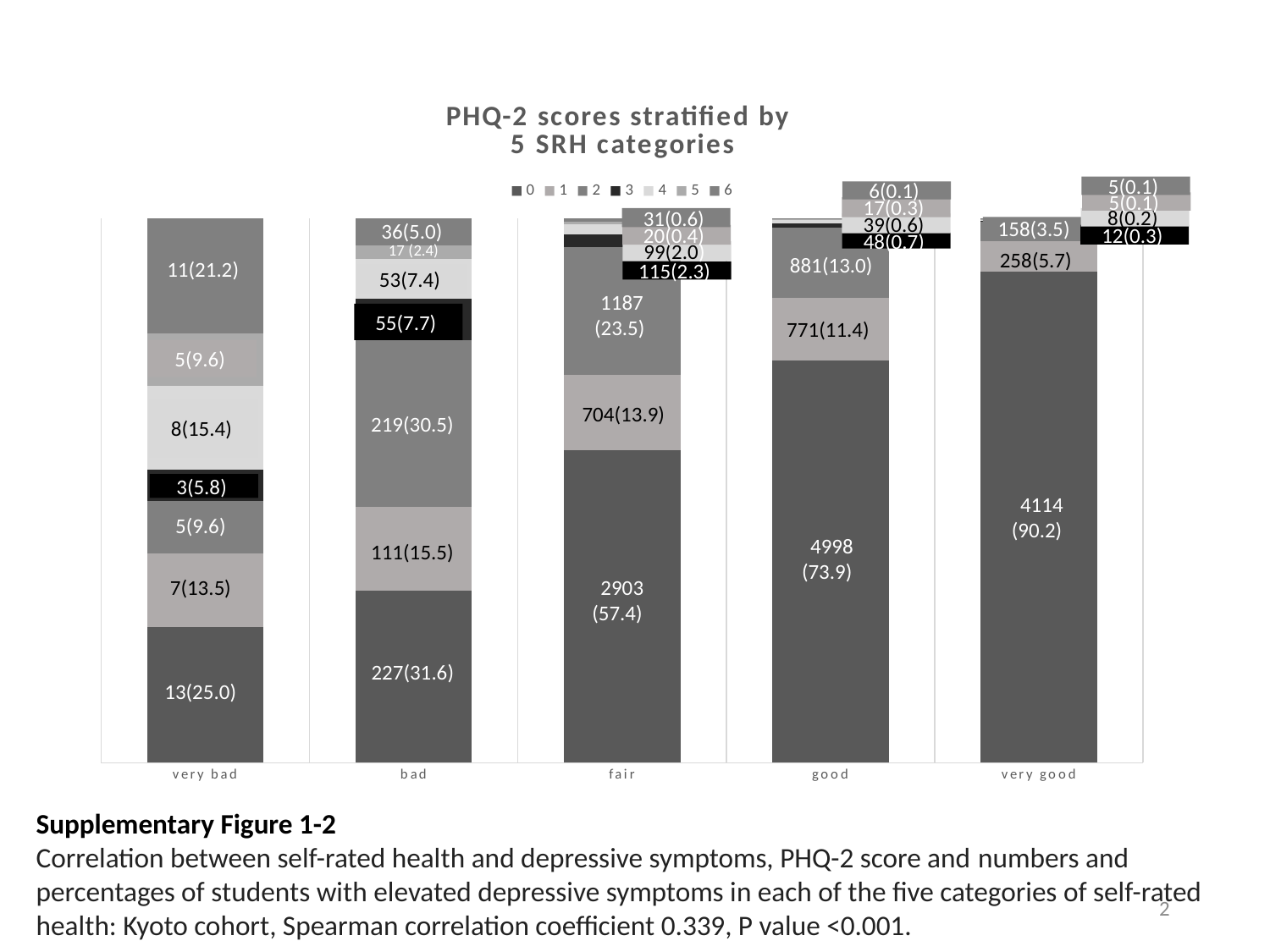

### Chart: PHQ-2 scores stratified by
5 SRH categories
| Category | 0 | 1 | 2 | 3 | 4 | 5 | 6 |
|---|---|---|---|---|---|---|---|
| very bad | 13.0 | 7.0 | 5.0 | 3.0 | 8.0 | 5.0 | 11.0 |
| bad | 227.0 | 111.0 | 219.0 | 55.0 | 53.0 | 17.0 | 36.0 |
| fair | 2903.0 | 704.0 | 1187.0 | 115.0 | 99.0 | 20.0 | 31.0 |
| good | 4998.0 | 771.0 | 881.0 | 48.0 | 39.0 | 17.0 | 6.0 |
| very good | 4114.0 | 258.0 | 158.0 | 12.0 | 8.0 | 5.0 | 5.0 |5(0.1)
6(0.1)
5(0.1)
17(0.3)
31(0.6)
8(0.2)
39(0.6)
158(3.5)
36(5.0)
12(0.3)
20(0.4)
48(0.7)
881(13.0)
11(21.2)
99(2.0)
258(5.7)
115(2.3)
53(7.4)
1187
(23.5)
771(11.4)
55(7.7)
5(9.6)
704(13.9)
219(30.5)
8(15.4)
3(5.8)
4114
(90.2)
5(9.6)
111(15.5)
4998
(73.9)
7(13.5)
2903
(57.4)
227(31.6)
13(25.0)
Supplementary Figure 1-2
Correlation between self-rated health and depressive symptoms, PHQ-2 score and numbers and percentages of students with elevated depressive symptoms in each of the five categories of self-rated health: Kyoto cohort, Spearman correlation coefficient 0.339, P value <0.001.
2
